# Association of cytokine patterns and clinical/laboratory parameters, medication and periodontal burden in patients with rheumatoid arthritis (RA)

**DOI:** 10.1007/s10266-020-00517-9

**Published:** 2020-04-16

**Authors:** S. Patschan, L. Bothmann, D. Patschan, E. Henze, G. Schmalz, O. Ritter, D. Ziebolz

**Affiliations:** 1 Department of Cardiology, Angiology and Nephrology, Klinikum Brandenburg, Medizinische Hochschule Brandenburg, Hochstraße 29, 14770 Brandenburg, Germany; 2 Clinic of Nephrology and Rheumatology, Universitätsmedizin Göttingen, Göttingen, Germany; 3 Department of Cariology, Endodontology and Periodontology, University Leipzig, Leipzig, Germany

**Keywords:** Cytokines, Rheumatoid arthritis, Periodontal health, Disease activity, Tooth loss

## Abstract

To evaluate serum levels of the following cytokines in rheumatoid arthritis subjects with periodontal disease: Interleukin-6, -10, -17, and -23. Patients with rheumatoid arthritis frequently suffer from periodontal disease. Both diseases partly result from a dysregulated immune response. The current study aimed to quantify Interleukin-6, -10, -17, and -23 levels in rheumatoid arthritis. It should be investigated if the periodontal disease would have additional modifying effects. A total of 157 patients were included. Serum levels of IL-6, -10, -17, and -23 were measured by ELISA. Serum IL-10 increased with longer duration of morning stiffness and with higher rheumatoid factor and anti-cyclic citrullinated peptide titres. IL-10 was also elevated with longer duration of prednisolone (< 5 mg daily) and leflunomide therapy. Subjects with lower erythrocyte sedimentation rate/longer leflunomide therapy displayed more missing teeth/more clinical attachment loss. IL-17 was higher in subjects with fewer missing teeth if the following criteria were fulfilled: shorter prednisolone (< 5 mg) and methotrexate therapy, more swollen joints, longer morning stiffness. IL-23 finally was increased in subjects with higher rheumatoid factor and in those with higher periodontal probing depth/clinical attachment loss in the following situations: lower rheumatoid factor and shorter leflunomide therapy. Subjects suffering from dental/periodontal burden show an aberrant systemic cytokine availability of serum IL-6, IL-10, IL-17 and IL-23 related to disease activity and medication. This examination underlines the complexity of potential interactions between disease activity and medication related to periodontal burden.

## Introduction

Rheumatoid arthritis (RA) causes synovial inflammation, subsequently followed by joint destruction and disablement if treatment has been initiated too late and/or is inefficient. Besides joint involvement, the disease may affect inner organs such as vessels and heart in a specific manner. In addition, RA is associated with increased cardiovascular risk [1]. Finally, affected individuals may significantly suffer from periodontal disease. Epidemiological associations between periodontal disease (PD) and RA have reliably been documented [2–6] and according to current hypotheses, periodontitis is thought to enhance RA-related autoantibody-production, even years before RA evolves clinically [7]. Both disorders, RA and PD exhibit similarities regarding their pathogenesis. Firstly, an inadequate host immune response causes soft tissue inflammation possibly followed by chronic and irreversible damage of hard tissues such as cartilage and bone. Secondly, obesity and smoking have been identified as potential risk factors in both situations [8–11]. Another hallmark of the two diseases is dysregulation of the local (periarticular/periodontal) and systemic cytokine production, to a significant extent reflecting the aberrant immune response. Respective data have been presented in RA [12] and PD [13–15]. Particularly in RA, local and systemic cytokine profiling ultimately facilitated the introduction of new therapeutics, the biologics, which act in a more selective manner than conventional disease-modifying anti-rheumatic drugs (cDMARDs) such as methotrexate and leflunomide. These medications show also a certain potential to influence periodontal inflammation and disease burden [16]. The current study aimed to examine whether the cytokine level of Interleukin-6, -10, -17, and -23 in serum would be associated with RA-related clinical and laboratory findings. Furthermore, it should be investigated if clinical signs of PD would have an additional modifying effect on these associations. The study was part of a large-scaled project on periodontal health in RA, several associated analyzes were published previously [16].

## Patients and methods

### Patients

All patients were recruited from the Clinic of Nephrology and Rheumatology and from the Department of Preventive Dentistry, Periodontology, and Cariology of the University Medical Center Goettingen (Goettingen, Germany). The subjects analyzed in this study were evaluated for numerous parameters of disease activity and clinical parameters of oral health (dental and periodontal). Other aspects of this particular cohort were published previously [17, 18]. The study was approved by the local ethics committee of the Medical Center and all participants signed written consent. Inclusion criteria: diagnosis of rheumatoid arthritis according to the clinical symptoms in conjunction with laboratory and/or radiographic findings or by applying the 2010 revised ACR (American College of Rheumatology)-/EULAR (European League Against Rheumatism)-classification criteria of the disease [19]; age > 18 and < 90 years; gender: male or female; disease-modifying anti-rheumatic drug (DMARD) therapy with either conventional drugs (cDMARDs—methotrexate, leflunomide, sulfasalazine; alone or in combination) and/or biologic drugs (bDMARDs—anti-TNF-alpha such as adalimumab, certolizumab pegol, etanercept, golimumab, infliximab; anti-IL6 tocilizumab; anti-CD20—rituximab). Exclusion criteria: uncontrolled systemic infection, uncontrolled tumor disease, dialysis dependence, rejection to participate (also if declared at any later timepoint). Positive findings of rheumatic factor (RF) and/or anti-cyclic citrullinated peptide (anti-CCP) (low titre: ≤ threefold of the upper threshold; high titre > threefold of the upper threshold) defined a patient as seropositive. Subjects were regularly followed-up at the inpatient clinic of the Medical Center. In addition to DMARD therapy, individuals received variable doses of prednisolone and of non-steroidal anti-inflammatory drugs (NSAIDs) if needed. Disease activity was quantified using the DAS28-ESR (0–3.2: inactive disease; 3.2–5.1: moderate activity; above 5.1: active disease) [20] and by additional serological testing (c-reactive protein).

### Dental and periodontal examination

Oral examination of all subjects was performed in a standardized and controlled manner by experienced dentists. The examination was assessed visually. The parameter of dental health was the respective number of missing teeth (M-T). Wisdom teeth (third molars) were excluded from the assessment. Periodontal examination included the following parameters: periodontal probing depth (PPD) and clinical attachment loss (CAL) in mm. Based on the definition of the AAP/CDC, the severity of periodontitis was classified into none/mild (neither severe nor moderate periodontitis), moderate (≥ 2 interproximal sites with AL ≥ 4 mm [not on the same tooth] or ≥ 2 interproximal sites with PD ≥ 5 mm) or severe periodontitis (≥ 2 interproximal sites with an attachment loss (AL) ≥ 6 mm [not on the same tooth] and ≥ 1 interproximal site with PD ≥ 5 mm.

### Serological analyses

Serum samples of subjects were quantified for the following cytokines: IL-6, -10, -17, and -23. Analyses were performed in the department of clinical chemistry of the Medical Center according to standardized procedures.

### Statistical analyses

The analysis was separated into two subsections. The first subsection evaluated cytokine levels in relation to four clinical categories, four laboratory findings, and to five therapy-related categories. The clinical categories were: DAS28, number of swollen joints (NSJ), number of painful joints (NPJ), and morning stiffness in minutes. The laboratory findings were: titre of RF, titre of anti-CCP, CRP, and erythrocyte sedimentation rate (ESR) after hour 1. The five therapy-related categories were: daily prednisolone dose ≤ 5 mg, daily prednisolone dose > 5 mg, methotrexate therapy, leflunomide therapy, and anti-TNF-alpha therapy. In all mentioned analyses, the respective means served as cut-off values. The second subsection evaluated cytokine levels in relation to the three parameters of dental and periodontal health (M-T, PPD, CAL). In every analysis, two groups were defined: group one—subjects below the mean of the respective category (e.g. DAS28, RF titre, duration of methotrexate therapy in months) versus group two—subjects ≥ the mean. Every group was further divided into two subgroups, according to the mean of an individual dental/periodontal health parameter (e.g. M-T, PPD). All data are presented as mean ± SEM. Differences between two groups were evaluated by the student’s *t* test, differences between three or more groups using ANOVA. Significance was postulated if *p*-values were below 0.05.

## Results

### Patients

A total of 157 patients (129 female, 28 male) with a mean age of 60.5 ± 0.8 years were included in the current study. All patients´characteristics are summarized in Table 1.

**Table 1:** Baseline characteristics of all subjects included

Variable	Results
Age (years)	60.5 ± 0.8
Gender	129 females, 28 males
Mean duration of the disease (DOD—years)	10.2 ± 1.1
Mean number of swollen joints (NSJ)	3.1 ± 0.3
Mean number of painful joints (NPJ)	6.4 ± 0.5
Mean duration of morning stiffness (minutes)	37.8 ± 3.8
Seropositivity (detection of RF and/or anti-CCP) %)	52
Mean ESR in hour 1 (mm)	12.6 ± 1
mean CRP (mg/l)	2.7 ± 0.1
*Immunosuppression at the time of study inclusion (n)*	
Methotrexate	87
Leflunomide	40
Anti-TNF-alpha	28
Others (sulfasalazine, hydroxychloroquine, abatacept, tocilizumab, rituximab)	54
Daily prednisolone dose ≤ 5 mg	106
Daily prednisolone dose > 5 mg	17
*Parameters of dental and periodontal health*	
M-T	6.4 ± 0.46
CAL	3.1 ± 0.1
PPD	2.9 ± 0.1

### Serological characteristics

The mean overall serum levels were: IL-6—33.9 ± 9.8 pg/ml, IL-10—29.9 ± 11.7 pg/ml, IL-17—13.1 ± 1.8 pg/ml, and IL-23—69.9 ± 19.3 pg/ml. The following sections will exclusively name numerical results that met the criteria of statistical significance (*p* < 0.05). All other results will either appear in the figures or tables.

### Subsection one

#### Clinical categories

**Fig. 1:**
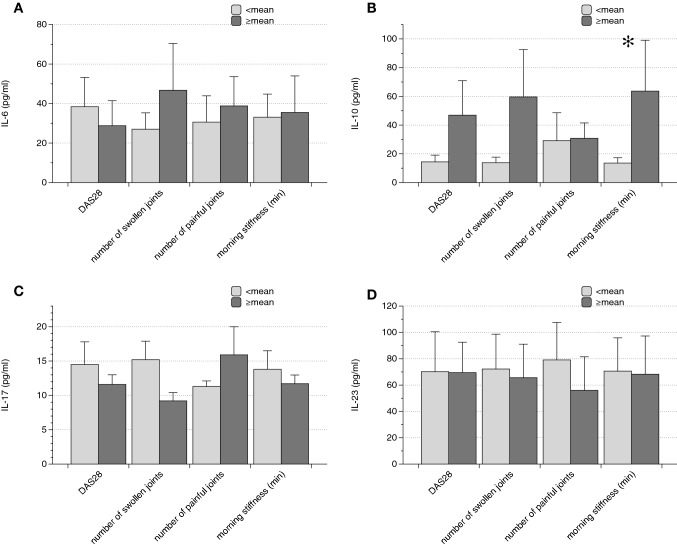
Cytokine levels in relation to disease activity (DAS28-ESR—DAS28), number of swollen and painful joints and morning stiffness. As detailed in the results section, the respective means were employed as cut-offs. The only significant difference identified was higher serum IL-10 in subjects with longer duration of morning stiffness (Data as mean ± SEM, *p < 0.05)

The serum concentrations of IL-10 were higher in individuals with longer duration of morning stiffness (13.5 ± 3.7 vs. 63.7 ± 35.4 pg/ml, *p* = 0.04). Further statistically significant differences for the examined cytokines were not found (Fig. 1).

#### Laboratory findings

**Fig. 2:**
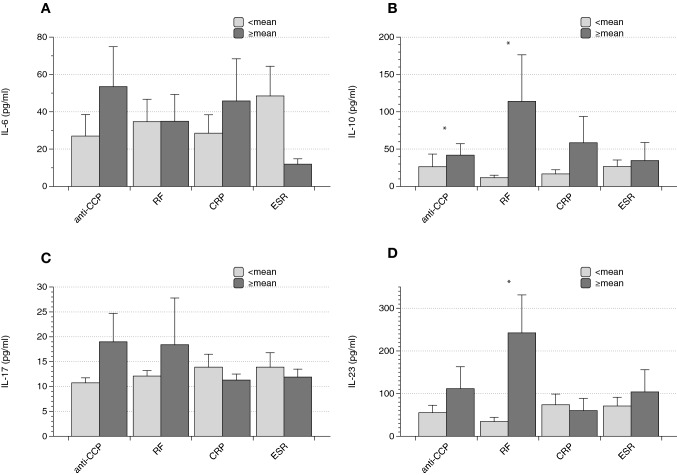
Cytokines and laboratory findings. Patients were evaluated for rheumatoid factor (RF) and anti-CCP, in addition humoral inflammation was characterized by measuring CRP and ESR. The respective means once again served as cut-offs. Interleukin-10 was higher in subjects with elevated RF and elevated anti-CCP, Interleukin-23 was significantly higher in patients with ≥ the mean RF titer (Data as mean ± SEM, *p < 0.05)

For IL-10, two differences were identified: subjects with lower than the means of anti-CCP and RF displayed lower serum concentrations (anti-CCP 26.4 ± 16.9 vs. 41.9 ± 15.4 pg/ml, *p* < 0.0001; RF 11.7 ± 3.2 vs. 114.5 ± 62.3 pg/ml, *p* = 0.01). Furthermore, IL-23 was significantly higher in subjects with higher RF (RF 34.5 ± 9.8 vs. 242.5 ± 89.2 pg/ml, *p* < 0.0001). Further significant findings could not be detected (Fig. 2).

#### Therapy-related categories

**Fig. 3:**
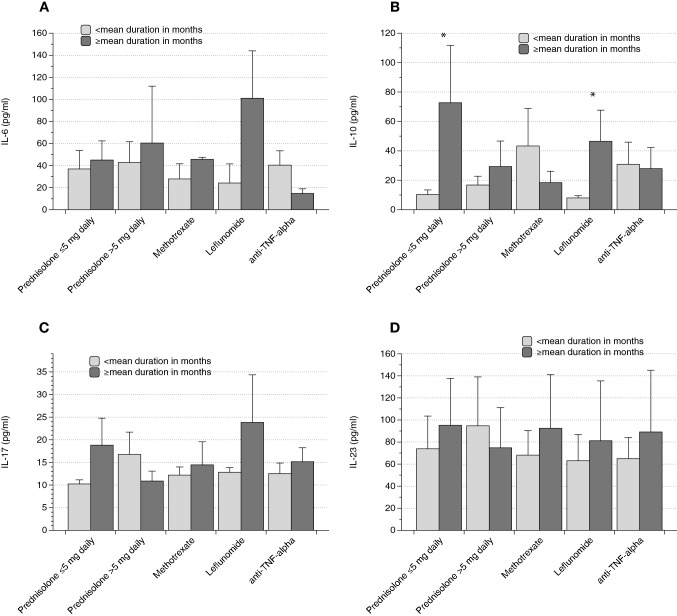
Treatment characteristics and cytokines. The only difference occurred in Leflunomide-treated subjects: serum IL-10 was higher with longer duration of therapy. All other groups did not differ significantly. The respective duration of drug administration in months was evaluated and the means were used as cut-offs (Data as mean ± SEM, *p < 0.05)

Subjects receiving less than 5 mg Prednisolone daily showed higher serum IL-10 with longer duration of therapy (10.3 ± 3.1 vs. 72.7 ± 39 pg/ml, *p* = 0.03), the same observation was made in individuals undergoing Leflunomide treatment (8 ± 1.5 vs. 46.5 ± 21.1 pg/ml, *p* = 0.01). There were no differences in other categories (Fig. 3).

### Subsection two

#### Missing teeth (M-T)

Subjects with more than the average NSJ and those with longer than the average duration of morning stiffness (DOMS) displayed higher IL-17 if fewer teeth were missing (11.4 ± 1.4 vs. 5.7 ± 1.8 pg/ml; *p* = 0.02 and 13.8 ± 1.3 vs. 7 ± 1.7; *p* = 0.008). Serum IL-10 was higher in the ‘below mean ESR’ subgroup if more teeth were absent (11.9 ± 4.2 vs. 48.4 ± 22.2; *p* = 0.04). Individuals with a lower duration of therapy (DUO) with daily prednisolone < 5 mg and those below the mean methotrexate DUO showed higher IL-17 with fewer numbers of M-T (11.3 ± 1.3 vs. 7.1 ± 1.3; *p* = 0.03; 16.1 ± 2.6 vs. 7 ± 1.4; *p* = 0.01) (Table 2).

**Table 2:** Cytokine analysis in relation to the numbers of missing teeth and subdivided by clinical, serological, and therapy-related parameters

Cytokine	Clinic	*p*	Serology	*p*	Therapy	*p*
	* < mean DOMS*		* < mean ESR*		* < mean DUO LEF*	
< vs. ≥ mCAL IL-6	33.7 ± 16.7 vs. 32.4 ± 16.3	0.95	52.1 ± 21.4 vs. 42 ± 23.1	0.76	48.3 ± 34.1 vs. 7.6 ± 1.6	0.29
< vs. ≥ mCAL IL-10	9.9 ± 1.6 vs. 18.2 ± 8	0.27	17.1 ± 7.9 vs. 36.4 ± 16.7	0.26	7.1 ± 1.6 vs. 8.3 ± 2.1	0.63
< vs. ≥ mCAL IL-17	13 ± 2.3 vs. 14.8 ± 5.3	0.73	11.6 ± 1.7 vs. 18.2 ± 7.6	0.28	13.5 ± 1.3 vs. 12.2 ± 1.7	0.38
< vs. ≥ mCAL IL-23	61.3 ± 35.5 vs. 82.3 ± 35.7	0.68	31.6 ± 7.4 vs. 69.6 ± 42	0.25	9.8 ± 2.9 vs. 104 ± 37.1	**0.02**
	* ≥ mean DOMS*		* ≥ mean ESR*		* ≥ mean DUO LEF*	
< vs. ≥ mCAL IL-6	55.1 ± 33.2 vs. 12.5 ± 4.7	0.25	12.9 ± 4.8 vs. 11.3 ± 3.9	0.84	85.3 ± 58.4 vs. 127 ± 68.2	0.65
< vs. ≥ mCAL IL-10	86.1 ± 62.7 vs. 37.6 ± 20.3	0.49	74.6 ± 65.9 vs. 14.2 ± 6.4	0.36	9.2 ± 1.6 vs. 106 ± 45.2	**0.01**
< vs. ≥ mCAL IL-17	9.9 ± 1.6 vs. 13.8 ± 1.8	0.12	13 ± 3.5 vs. 11.1 ± 1.5	0.75	14.3 ± 6.9 vs. 39 ± 25.1	0.27
< vs. ≥ mCAL IL-23	77.2 ± 47.9 vs. 57.8 ± 28.5	0.74	149 ± 91.6 vs. 78.2 ± 31.7	0.61	32.6 ± 10.6 vs. 159 ± 141	0.27

*mM-T* mean M-T, *NSJ* number of swollen joints, *NPJ* number of painful joints, *DUO* duration of therapy

Differences that met the criteria of statistical significance are highlighted in white (Data as mean ± SEM)

#### Periodontal probing depth (PPD)

Individuals with lower RF titre and subjects with lower than the average duration of leflunomide therapy showed higher serum IL-23 with higher than average PPD (mPPD) (16.3 ± 2.7 vs. 57.2 ± 21.3; *p* = 0.03 and 9.2 ± 2.7 vs. 154 ± 54; *p* = 0.002) (Table 3).

**Table 3:** Cytokine analysis in relation to the periodontal probing depth (PPD) and subdivided by clinical, serological, and therapy-related parameters

Cytokine	Clinic	*p*	Serology	*p*	Therapy	*p*
	* < mean DAS28*		* < mean RF*		* < mean DUO P < 5*	
< vs. ≥ mM-T IL-6	30.6 ± 13.7 vs. 58.1 ± 40.2	0.4	31.8 ± 15.2 vs. 39.5 ± 21.6	0.76	36.2 ± 24.7 vs. 46.7 ± 37	0.8
< vs. ≥ mM-T IL-10	11.6 ± 4.1 vs. 21.6 ± 13.4	0.34	7.2 ± 0.9 vs. 20.6 ± 8.6	0.05	7.4 ± 1.4 vs. 15.6 ± 9.3	0.3
< vs. ≥ mM-T IL-17	17.3 ± 4.6 vs. 7.5 ± 1.7	0.18	13.6 ± 1.4 vs. 9.9 ± 2	0.15	11.3 ± 1.3 vs. 7.1 ± 1.3	**0.03**
< vs. ≥ mM-T IL-23	91.8 ± 42.5 vs. 16 ± 4.2	0.26	46.5 ± 15.9 vs. 16.8 ± 3	0.15	119 ± 59.6 vs. 18 ± 4.4	0.17
	* ≥ mean NSJ*		* ≥ mean ACPA*		* ≥ mean DUO P ≥ 5*	
< vs. ≥ mM-T IL-6	37.6 ± 27.3 vs. 60.8 ± 43.6	0.63	37.1 ± 17.6 vs. 69.8 ± 39.4	0.45	102 ± 92.9 vs. 12.3 ± 5.8	0.49
< vs. ≥ mM-T IL-10	35.7 ± 19.7 vs. 96.7 ± 77.8	0.36	19.1 ± 9.7 vs. 64.7 ± 28.4	0.14	13.6 ± 4.9 vs. 58.3 ± 53.9	0.3
< vs. ≥ mM-T IL-17	11.4 ± 1.4 vs. 5.7 ± 1.8	**0.02**	26.9 ± 11 vs. 11 ± 1.9	0.17	10.8 ± 0.9 vs. 6.3 ± 3.1	0.14
< vs. ≥ mM-T IL-23	50.5 ± 17.2 vs. 89 ± 59.5	0.46	97.2 ± 59.3 vs. 126 ± 86	0.78	79.8 ± 59 vs. 16.3 ± 6	0.45
	* < mean NPJ*		* < mean CRP*		* < mean DUO MTX*	
< vs. ≥ mM-T IL-6	23.3 ± 11 vs. 46.8 ± 32.4	0.39	17.5 ± 19.2 vs. 41.9 ± 21.6	0.25	33.9 ± 24.6 vs. 21 ± 8.5	0.66
< vs. ≥ mM-T IL-10	8 ± 1.2 vs. 76 ± 57.3	0.08	11.3 ± 11 vs. 24.9 ± 12.4	0.26	9 ± 1.9 vs. 76.4 ± 61.2	0.2
< vs. ≥ mM-T IL-17	12.9 ± 1.75 vs. 7.7 ± 1.3	0.06	17.8 ± 1.8 vs. 9.9 ± 2	0.16	16.1 ± 2.6 vs. 7 ± 1.4	**0.01**
< vs. ≥ mM-T IL-23	87.6 ± 35.9 vs. 60.5 ± 44.1	0.65	84.3 ± 40.7 vs. 72.8 ± 45.8	0.82	50.4 ± 20.8 vs. 75.2 ± 47	0.6
	* < mean DOMS*		* < mean ESR*		* < mean DUO LEF*	
< vs. ≥ mM-T IL-6	26.3 ± 12.6 vs. 42.2 ± 22.2	0.52	45.4 ± 20.7 vs. 57.1 ± 30.2	0.74	29.5 ± 22.2 vs. 6.5 ± 3.9	0.66
< vs. ≥ mM-T IL-10	11.2 ± 3.8 vs. 17 ± 7.3	0.44	11.9 ± 4.2 vs. 48.4 ± 22.2	**0.04**	8.4 ± 1.8 vs. 6.2 ± 3.6	0.63
< vs. ≥ mM-T IL-17	16.6 ± 4.3 vs. 9.7 ± 2.1	0.21	17.3 ± 4.7 vs. 9.3 ± 1.7	0.22	12.6 ± 1.3 vs. 10.5 ± 3.5	0.52
< vs. ≥ mM-T IL-23	104 ± 41.5 vs. 21.1 ± 4.5	0.1	51.5 ± 25.6 vs. 40.6 ± 11.6	0.76	81.4 ± 29.6 vs. 14.2 ± 10.2	0.34
	* ≥ mean DOMS*		* ≥ mean ESR*		* ≥ mean DUO LEF*	
< vs. ≥ mM-T IL-6	40.4 ± 25.8 vs. 24.3 ± 13.3	0.69	10.5 ± 2.1 vs. 14.5 ± 6	0.53	116 ± 57.3 vs. 89.4 ± 67.1	0.77
< vs. ≥ mM-T IL-10	35.2 ± 18.6 vs. 128 ± 106	0.22	8 ± 2 vs. 74 ± 57	0.25	27 ± 19.7 vs. 63.2 ± 35.8	0.41
< vs. ≥ mM-T IL-17	13.8 ± 1.3 vs. 7 ± 1.7	**0.008**	12.5 ± 1.5 vs. 10.9 ± 3.1	0.65	41.1 ± 21 vs. 9 ± 3.6	0.13
< vs. ≥ mM-T IL-23	51 ± 20.8 vs. 107 ± 82.3	0.37	99 ± 38.7 vs. 128 ± 80.1	0.74	139 ± 117 vs. 31.2 ± 11	0.34

*mPPD* mean PPD, *NSJ* number of swollen joints, *NPJ* number of painful joints, *DUO* duration of therapy

Differences that met the criteria of statistical significance are highlighted in white (Data as mean ± SEM)

#### Clinical attachment loss (CAL)

Subjects with lower than the average duration of leflunomide therapy showed higher serum IL-23 with higher than average CAL (9.8 ± 2.9 vs. 104 ± 37.1; *p* = 0.02) and individuals with higher than (or equal to) the average duration of leflunomide therapy displayed higher serum IL-10 (9.2 ± 1.6 vs. 106 ± 45.2; *p* = 0.01) (Table 4).

**Table 4:** Cytokine analysis in relation to the clinical attachement loss (CAL) and subdivided by clinical, serological, and therapy-related parameters

Cytokine	Clinic	*p*	Serology	*p*	Therapy	*p*
	* < mean DAS28*		* < mean RF*		* < mean DUO P < 5*	
< vs. ≥ mPPD IL-6	40.5 ± 22.3 vs. 36.3 ± 19.7	0.89	46 ± 19.1 vs. 20.7 ± 12.6	0.29	40.4 ± 26.8 vs. 41.3 ± 36	0.98
< vs. ≥ mPPD IL-10	8 ± 1.6 vs. 21.5 ± 9.7	0.15	8.5 ± 1.4 vs. 16.5 ± 7.1	0.22	7 ± 1.1 vs. 17 ± 9.4	0.22
< vs. ≥ mPPD IL-17	12.1 ± 2.6 vs. 17 ± 6.4	0.47	12.1 ± 12 vs. 12.1 ± 0.83	0.99	8.3 ± 1.2 vs. 11.7 ± 1.4	0.08
< vs. ≥ mPPD IL-23	62.4 ± 47.7 vs. 78.7 ± 37.2	0.79	16.3 ± 2.7 vs. 57.2 ± 21.3	**0.03**	71 ± 58.4 vs. 105 ± 40.3	0.65
	* < mean DOMS*		* < mean ESR*		* < mean DUO LEF*	
< vs. ≥ mPPD IL-6	31.8 ± 15.5 vs. 35.1 ± 18	0.89	41 ± 17.7 vs. 59.1 ± 29.4	0.58	36.8 ± 31.2 vs. 9.4 ± 2.2	0.48
< vs. ≥ mPPD IL-10	9.3 ± 1.4 vs. 19.7 ± 8.8	0.17	26.1 ± 12.1 vs. 21 ± 9.7	0.75	6.8 ± 1.5 vs. 10.7 ± 3.2	0.24
< vs. ≥ mPPD IL-17	12.2 ± 2.2 vs. 16.1 ± 5.8	0.47	11.9 ± 2.1 vs. 16.8 ± 6.4	0.41	11.8 ± 1.5 vs. 14.2 ± 1.5	0.29
< vs. ≥ mPPD IL-23	52.3 ± 32.8 vs. 97.3 ± 39.2	0.38	29 ± 7.2 vs. 67.7 ± 36.8	0.23	9.2 ± 2.7 vs. 154 ± 54	**0.002**

*mCAL* mean CAL, *NSJ* number of swollen joints, *NPJ* number of painful joints, *DUO* duration of therapy

Differences that met the criteria of statistical significance are highlighted in white (Data as mean ± SEM)

## Discussion

In this section, the results shall be discussed separately in relation to individual cytokines. Each paragraph will start with a short summary of the essential findings. Since our analyzes did not show any differences in serum IL-6 at all, we omitted to include IL-6 in the discussion section.

Interleukin-10 was higher in individuals with longer duration of morning stiffness and lower in subjects with lower anti-CCP and RF. Individuals with longer leflunomide therapy displayed higher serum IL-10, the cytokine was also higher in the ´below mean ESR´ subgroup if more teeth were absent. Interleukin-10 is widely regarded as anti-inflammatory regulator in RA. A newer experimental study revealed IL-10 production by myeloid-derived suppressor cells to be of critical importance for regulatory T cell induction in a murine model of rheumatoid inflammation [21]. The counterbalancing potency of IL-10 has, in fact, been known for many years. Meanwhile, therapeutic administration of IL-10 has been evaluated under clinical circumstances, the results were however not convincing [22, 23]. Our data somehow support the regulatory role of the cytokine in RA. Higher IL-10 levels in subjects with longer duration of morning stiffness suggest an endogenous counterbalancing mechanism in states of higher disease activity. Since seropositivity in RA is commonly regarded as a risk factor of disease progression [24], one may conclude that subjects with lower than the average RF/anti-CCP titres are less ‘in need’ of self-protection, for instance, mediated by IL-10. Higher IL-10 in patients undergoing leflunomide treatment for longer periods possibly reflects particular cytokine-stimulating effects of the substance per se. However, such an assumption is nothing but speculative at the moment since specific data on interrelationships between the drug and dynamic characteristics of IL-10 are not available yet. The last IL-10-related finding (elevated IL-10) showed more tooth loss in subjects with lower ESR in hour 1. Such a constellation is hardly explainable. Lower ESR may reflect anti-inflammatory effects of the cytokine but to conclude that IL-10 is involved in dental/periodontal destabilization appears untrustworthy to say the least.

In the following categories, Interleukin-17 was higher if fewer teeth were missing: higher NSJ, longer duration of morning stiffness (DOMS), lower duration of prednisolone therapy (DUO) (< 5 mg), and lower methotrexate DUO. Interleukin-17 acts as pro-inflammatory cytokine in certain rheumatic disease, namely in ankylosing spondylitis and psoriatic arthritis [25, 26]. Antibody-mediated neutralization of IL-17 is an established anti-progressive treatment option in both disorders. Although the cytokine plays a critical role in the pathogenesis of RA as well [27], anti-IL-17 treatment [28] has not been established in the management of the disease so far. Joint involvement (NSJ) and morning stiffness are reliable parameters of disease activity. Lower prednisolone dosing, on the other hand, is exclusively feasible in subjects with lower RA activity. Therefore, it is once impossible to propose whether IL-17-related immunoreactions would be related to dental health in general. The examined periodontal parameters were not associated with aberrant serum IL-17 in the current study, making its relevance for oral disease burden questionable.

Interleukin-23 was significantly higher in subjects in the ´above or equal to the mean RF titre´ category. Individuals with lower RF titre and subjects with lower leflunomide DUO showed higher serum IL-23 with higher PPD (mPPD). Subjects with lower leflunomide DUO displayed higher serum IL-23 with higher CAL. Dysregulated serum IL-23 in RA has been documented by others as well [29]. Andersen et al. identified increased serum IL-23 in early RA with lower levels after initiation of anti-TNF-alpha treatment [30]. However, a 2012 published clinical phase IIa trial, evaluating the efficacy of anti-IL12/-23 failed to show clinical improvement in RA [31]. Serum levels of the cytokine have been shown to increase in generalized aggressive periodontitis [32]. Thus, the cytokine should most likely be considered as pro-progressive on RA and periodontal disease. This assumption is in line with higher serum levels in subjects displaying higher RF titres. The two other observations fit into this particular concept as well. Subjects belonging to the subcategory ´lower leflunomide DUO´ displayed higher IL-23 in cases of more severe PPD and CAL. This seems in line with previously published results, showing leflunomide to be associated with the decreased periodontal burden compared to other RA-related medications [16]. Thereby, immunoreactions including IL-23 related pathways might be responsible for lower PD severity. However, comparably to the other cytokines evaluated in the current study, substantial mechanical conclusions can only be drawn prematurely.

Strengths and limitations: With 157 participants, the current study was able to include a large cohort of patients suffering from RA. Considering the heterogeneity within this complex patient group regarding medication and disease activity, the comprehensive analysis of the different subgroups are an interesting and new approach. Nevertheless, the current study has several limitations that must be addressed. Although several relevant cytokines were examined, IL-6 -, IL-10, IL-17, and IL-23 are just a small extract of the variety of RA-related cytokines. Furthermore, the considered parameters PPD, CAL and M-T reflect periodontal burden indeed but do not allow conclusions about the recent inflammation or PD activity. The comprehensive analysis of the different subgroups inhere a further limitation, as separation into so many subgroups results in a small sample size and thus in limited statistical power. Furthermore, the cross-sectional study design does not allow any causative conclusions; for this, large-scaled longitudinal examinations would be necessary. Thereby, the current study underlines again the high complexity of RA patients regarding disease activity, cytokine levels, medication and periodontal burden in a complex interaction.

## Conclusions

RA subjects suffering from dental/periodontal burden show an aberrant systemic cytokine availability regarding the examined cytokines IL-6, IL-10, IL-17 and IL-23 related to disease activity and medication. Although some results are in line with established concepts about the roles of certain cytokines (e.g. IL-10) in the pathogenesis of RA and of PD (e.g. IL-23), mechanistical or even therapeutical conclusions cannot be drawn based on the current study’s findings. The examination underlines the complexity of interactions between disease activity and medication related to periodontal burden.
